# Supplementary material for: Large-scale testing of antimicrobial lethality at single-cell resolution predicts mycobacterial infection outcomes
Source: Nat Microbiol. 2026 Jan 9;11(2):566–83. doi: 10.1038/s41564-025-02217-y (PMC12872448; doi:10.1038/s41564-025-02217-y)
Supplement: Supplementary file 1 — Supplementary Figs. 1–3 and Tables 1–4. [file 41564_2025_2217_MOESM1_ESM.pdf]

nature microbiology

Supplementary information https://doi.org/10.1038/s41564-025-02217-y

Large-scale testing of antimicrobial lethality at single-cell resolution predicts mycobacterial infection outcomes

In the format provided by the authors and unedited

SUPPLEMENTARY FIGURES

Supplementary Fig. 1

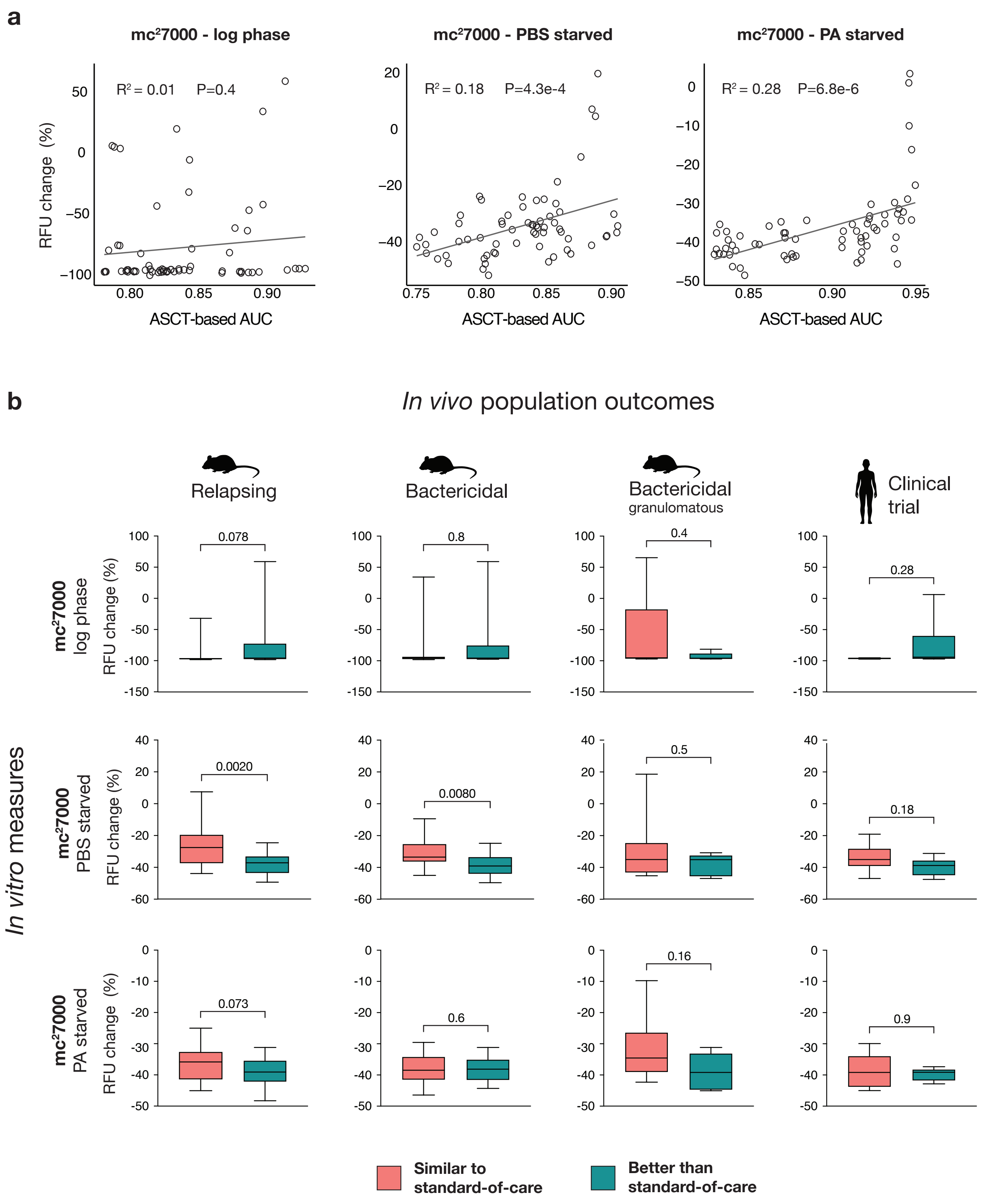

Supplementary Fig. 1. Association of resazurin-based killing assessments with ASCT and *in vivo* outcomes of *M. tuberculosis* drug regimens. (**a**) Correlation between resazurin-based killing (change in relative fluorescence units [RFU] from day 0 to 14) and ASCT-based killing of *M. tuberculosis* mc^{2}7000 (area under the time-kill curve). Correlation across 65 drug regimens was assessed using two-sided Pearson correlation. The solid line represents the linear regression fit. (**b**) Comparisons of resazurin-based killing of *M. tuberculosis* mc^{2}7000 (change in RFU from day 0 to 14) across *in vivo* classifications of drug regimens as similar-to-standard of care (SOC) or better-than-SOC, based on outcomes in relapsing mouse models (RMM), bactericidal mouse models (BMM) of common mouse strains, the granulomatous C3HeB/FeJ strain and clinical studies [32,33]. Groups were compared using a two-sided Mann-Whitney U test (P indicated). Boxplots show the median, interquartile range and total range (central line, box, and whiskers, respectively; RMM: n = 46, BMM common strains: n = 48, BMM C3HeB/FeJ: n = 15, clinical bactericidal activity: n = 14).

Supplementary Fig. 2

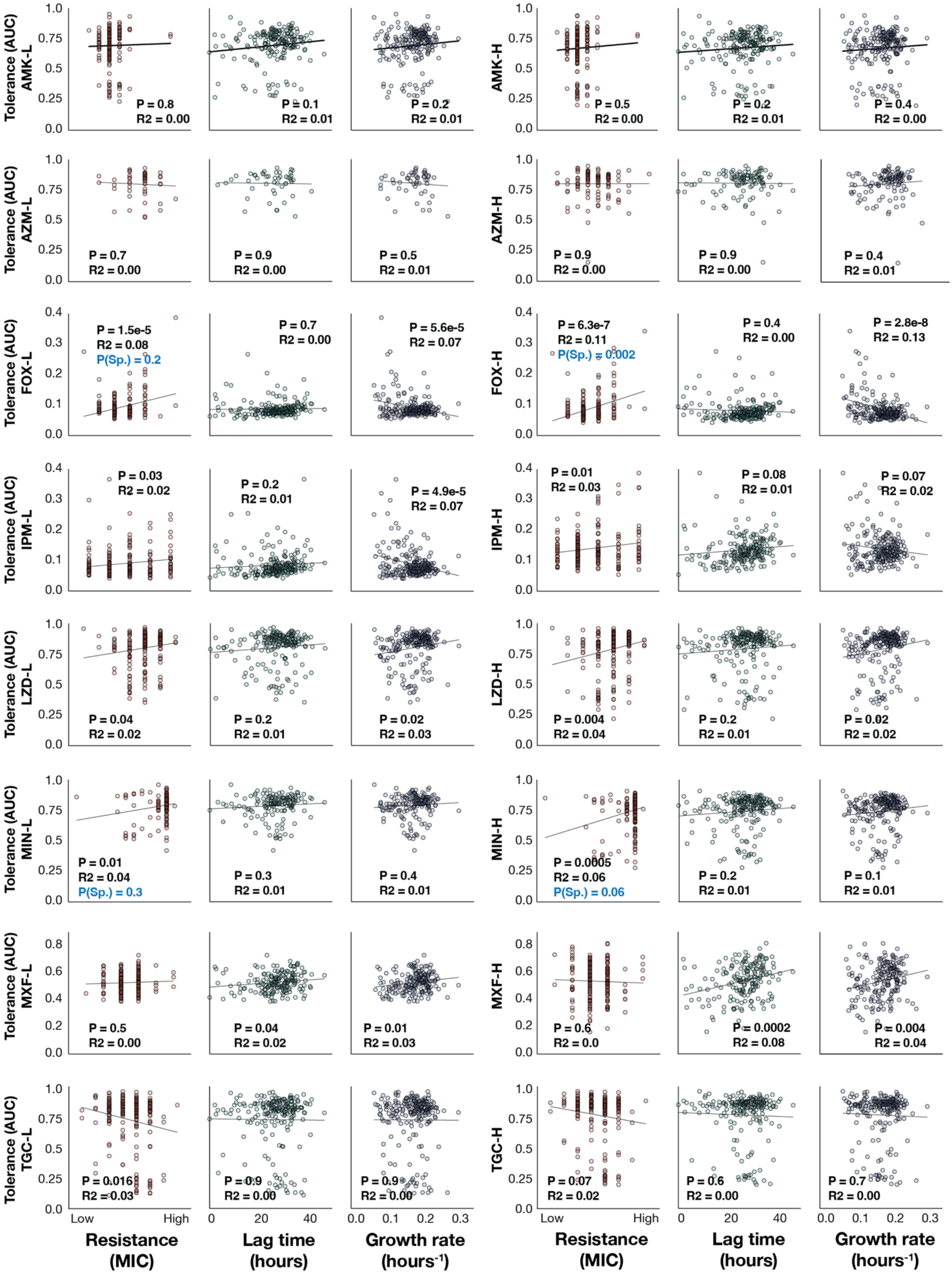

Supplementary Fig. 2. Correlations of *M. abscessus* drug tolerance with bacterial replication and drug resistance. Two-sided Pearson correlation analyses (in blue Spearman correlation) between drug tolerance and bacterial growth rate, lag time, and the corresponding minimum inhibitory concentration (MIC). AUC indicates the area under the kill curve, AMK amikacin, AZM azithromycin, FOX cefoxitin, IPM imipenem, LZD linezolid, MIN minocycline, MXF moxifloxacin, TGC tigecycline, L refers to low drug concentration, and H to high drug concentration.

Supplementary Fig. 3

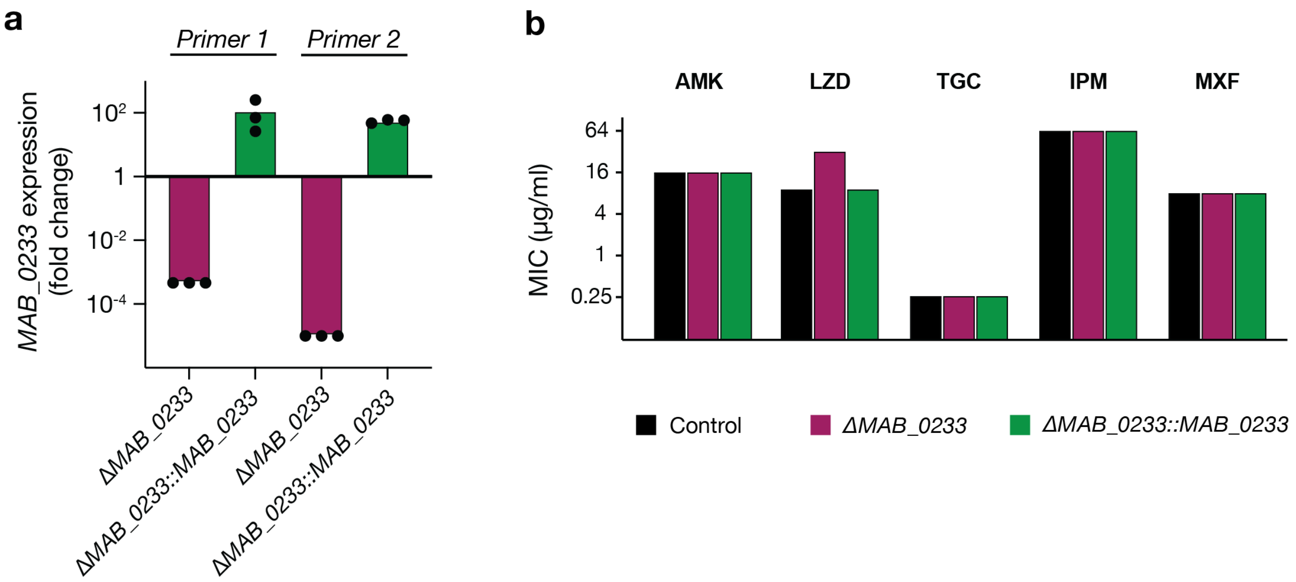

Supplementary Fig. 3. Gene expression and minimum inhibitory concentrations of *MAB_0233* mutants. (**a**) Log-fold change of *MAB_0233* expression in the *MAB*_0233 knockout and complemented strain compared to the control *M. abscessus* strain (three replicates each). (**b**) Minimum inhibitory concentrations (MICs) of control, knockout and complemented strains. AMK indicates amikacin, IPM imipenem, LZD linezolid, MXF moxifloxacin and TGC tigecycline.

SUPPLEMENTARY TABLES

	3-day live-cell fractions				7-day live-cell fractions			
	RMM	BMM	gBMM	Clinical	RMM	BMM	gBMM	Clinical
**H37Ra log phase**	0.018	0.023	1	0.23	0.048	0.055	0.8	0.9
**mc[2]7000 log phase**	0.9	1	0.06	0.7	0.9	0.7	0.19	0.14
**H37Ra PBS starved**	0.0013	0.015	0.13	0.043	0.0008	0.0043	0.099	0.029
**mc[2]7000 PBS starved**	0.0010	0.0035	0.0047	0.020	0.0017	0.011	0.0013	0.020
**mc[2]7000 PA starved**	0.0026	0.0070	0.028	0.0080	0.0026	0.010	0.040	0.020

Supplementary Table 1. Sensitivity analysis using ASCT-based live-cell fractions at day 3 and 7 after antibiotic exposure rather than AUCs. Statistical differences were assessed using a two-sided Mann-Whitney U test; corresponding P values are shown.

Model	AUC	Sensitivity	Specificity	PPV	NPV	F1_Score
Imipenem tolerance	0.68	0.59	0.79	0.85	0.67	0.69
Clarithromycin MIC	0.69	0.62	0.70	0.82	0.66	0.68
Combined MIC/tolerance	0.78	0.67	0.80	0.88	0.73	0.75

Supplementary Table 2. Performance of *M. abscessus* drug resistance (clarithromycin MIC) and drug tolerance (imipenem at low concentration) phenotypes for predicting clinical outcomes alone and in combination. AUC: area under the ROC-curve; NPV: negative predictive value; PPV: positive predictive value.

	AMK toleran. (AUC)	AZM tolerance (AUC)	FOX tolerance (AUC)	IPM tolerance (AUC)	LZD tolerance (AUC)	MIN tolerance (AUC)	MXF tolerance (AUC)	TGC tolerance (AUC)	Resistance - single feature
**AMK resistance (MIC)**	0.643 (0.617, 0.670)	0.640 (0.537, 0.743)	0.663 (0.644, 0.682)	0.696 (0.675, 0.717)	0.638 (0.615, 0.661)	0.631 (0.581, 0.682)	0.506 (0.450, 0.562)	0.636 (0.605, 0.666)	0.525 (0.503, 0.547)
**AZM resistance (MIC)**	0.670 (0.641, 0.699)	0.589 (0.516, 0.661)	0.684 (0.663, 0.705)	0.731 (0.709, 0.753)	0.663 (0.639, 0.686)	0.696 (0.665, 0.726)	0.603 (0.562, 0.644)	0.680 (0.650, 0.710)	0.642 (0.618, 0.666)
**CLR resistance (MIC)**	0.737 (0.709, 0.766)	0.661 (0.584, 0.739)	0.730 (0.704, 0.755)	0.775 (0.757, 0.794)	0.727 (0.700, 0.754)	0.747 (0.717, 0.776)	0.680 (0.649, 0.712)	0.732 (0.710, 0.754)	0.692 (0.668, 0.715)
**FOX resistance (MIC)**	0.649 (0.624, 0.674)	0.636 (0.541, 0.731)	0.684 (0.658, 0.710)	0.702 (0.676, 0.727)	0.595 (0.555, 0.636)	0.600 (0.554, 0.646)	0.532 (0.491, 0.574)	0.582 (0.535, 0.628)	0.539 (0.516, 0.562)
**IPM resistance (MIC)**	0.647 (0.621, 0.672)	0.629 (0.560, 0.698)	0.698 (0.672, 0.725)	0.697 (0.672, 0.721)	0.603 (0.561, 0.645)	0.630 (0.589, 0.671)	0.565 (0.528, 0.602)	0.632 (0.592, 0.672)	0.580 (0.554, 0.606)
**LZD resistance (MIC)**	0.649 (0.622, 0.676)	0.621 (0.518, 0.724)	0.643 (0.621, 0.664)	0.672 (0.649, 0.695)	0.628 (0.601, 0.654)	0.561 (0.519, 0.603)	0.545 (0.488, 0.601)	0.606 (0.580, 0.631)	0.517 (0.503, 0.532)
**MIN resistance (MIC)**	0.674 (0.648, 0.700)	0.648 (0.590, 0.706)	0.663 (0.637, 0.689)	0.700 (0.675, 0.725)	0.625 (0.594, 0.656)	0.592 (0.549, 0.636)	0.521 (0.468, 0.575)	0.631 (0.608, 0.653)	0.529 (0.508, 0.549)
**MXF resistance (MIC)**	0.645 (0.621, 0.669)	0.764 (0.729, 0.799)	0.681 (0.655, 0.707)	0.705 (0.674, 0.736)	0.582 (0.546, 0.618)	0.538 (0.470, 0.607)	0.527 (0.479, 0.575)	0.564 (0.519, 0.608)	0.517 (0.487, 0.546)
**TGC resistance (MIC)**	0.667 (0.644, 0.690)	0.633 (0.562, 0.705)	0.640 (0.614, 0.667)	0.681 (0.656, 0.705)	0.608 (0.581, 0.635)	0.610 (0.551, 0.669)	0.610 (0.585, 0.635)	0.589 (0.551, 0.628)	0.542 (0.519, 0.566)
**Tolerance - single feature**	0.652 (0.630, 0.675)	0.540 (0.472, 0.608)	0.650 (0.627, 0.673)	0.677 (0.658, 0.697)	0.610 (0.584, 0.636)	0.558 (0.528, 0.589)	0.510 (0.481, 0.540)	0.612 (0.586, 0.637)	

Supplementary Table 3. Logistic regression models predicting clinical outcomes from drug resistance - tolerance combinations, with mean AUC-ROC values and 95% confidence intervals. AMK indicates amikacin, AZM azithromycin, FOX cefoxitin, IPM imipenem, LZD linezolid, MIN minocycline, MXF moxifloxacin and TGC tigecycline.

Item	Group	Supplier	Product Nr.
**Amikacin Difulsate**	Chemicals and antibiotics	Ambeed	A125426
**Anhydrotetracycline (HCl)**	Chemicals and antibiotics	Adipogen	CAY-10009542-250
**Azithromycin**	Chemicals and antibiotics	Ambeed	A175409
**Bedaquiline**	Chemicals and antibiotics	AmBeed	A193466
**Cefoxitin Sodium Salt**	Chemicals and antibiotics	Sigma-Aldrich	C4786
**Clofazimine**	Chemicals and antibiotics	AmBeed	A316630
**D-Pantothenic acid hemicalcium salt**	Chemicals and antibiotics	Merck/SigmaAldrich	P5155-100G
**Delamanid**	Chemicals and antibiotics	AmBeed	A493940
**Ethambutol Hydrochloride**	Chemicals and antibiotics	Adipogen	CAY-23713-1
**Glycerol extra pure 99+%**	Chemicals and antibiotics	FisherScientific	10296200
**Imipenem Monohydrate**	Chemicals and antibiotics	Ambeed	A103901
**Isoniazid**	Chemicals and antibiotics	AmBeed	A432881
**Kanamycin A**	Chemicals and antibiotics	Adipogen	CAY-15321-5
**Linezolid**	Chemicals and antibiotics	AmBeed	A360550
**Methylsulfoxid, 99.8+%, ExtraPure DMSO**	Chemicals and antibiotics	FisherScientific	10562841
**Minocycline Hydrochloride**	Chemicals and antibiotics	Ambeed	A259020
**Moxifloxacin Hydrochloride**	Chemicals and antibiotics	AmBeed	A250011
**Pretomanid**	Chemicals and antibiotics	AmBeed	A204906
**Propidium iodide**	Chemicals and antibiotics	ThermoFisher	P3566
**Pyrazinamide**	Chemicals and antibiotics	FisherScientific	10484725
**Rifabutin**	Chemicals and antibiotics	AmBeed	A878035
**Rifampicin**	Chemicals and antibiotics	Adipogen	CAY-14423
**Rifapentin**	Chemicals and antibiotics	AmBeed	A444634
**SeaPrep AGAROSE Ultra-low Gelling Temperature**	Chemicals and antibiotics	RUWAG	LZ-50302
**SQ109**	Chemicals and antibiotics	Merck	SML1309
**Sterile PBS 10X, 1L**	Chemicals and antibiotics	Milian	91549
**Sutezolid (PNU 100180)**	Chemicals and antibiotics	Cayman	28395
**Tigecycline**	Chemicals and antibiotics	Ambeed	A175151
**Tween 80**	Chemicals and antibiotics	Merck	P8074-100ML
**Zeocin^t, 100 mg/ml in HEPES, sterile-filtered**	Chemicals and antibiotics	FisherScientific	J67140.8EQ
**BD BBL Middlebrook-OADC-Anreicherung**	Media	FisherScientific	11708173
**BD Mueller Hinton II Agar**	Media	SAP	9177901
**Middlebrook 7H11**	Media	ThomasScientific	C838M51
**Middlebrook 7H9 Broth Base**	Media	SigmaAldrich	M0178-500G
**Agarose, 100g (BioReagent, for molecular biology, low EEO)**	Molecular biology	SigmaAldrich	A9539
**DH5alpha Competent Cells, 10X100ul**	Molecular biology	ThermoFisher/Thermo Scientific	EC0112
**Direct-zol, DNA/RNA MiniPrep Plus w/ Zymo-Spin**	Molecular biology	Lucerna-Chem/Zymo Research	R2080
**dNTP 10mM (1ml)**	Molecular biology	BioConcept/Hangzhou Bioer Technology Co., Ltd.	BSA14S2B
**DreamTaq DNA Polymerase (5 U/¬µL)**	Molecular biology	FisherScientific/Thermo Scientific	EP0702
**GenElute Plasmid Miniprep Kit 70 Prep**	Molecular biology	Merck/SigmaAldrich	PLN70-1KT
**GeneRuler 1 kb DNA Ladder**	Molecular biology	ThermoFisher/Thermo Scientific	SM0311¬†¬†
**High-Capacity cDNA Reverse Transcription Kit**	Molecular biology	ThermoFisher/Applied Biosystems	A25741

**HindIII-HF, 50'000 units**	Molecular biology	BioConcept/New England Biolabs	R3104S
**KAPA HiFi HotStart ReadyMix - 1.25ml**	Molecular biology	Roche	KK2601
**Monarch PCR and DNA Cleanup Kit (5ug) 100 preps**	Molecular biology	BioConcept/New England Biolabs	T1034L
**PowerUp SYBR Green Master Mix**	Molecular biology	FisherScientific/Applied Biosystems	15350929
**PureLink DNA-Gel extraction and PCR CleanUp Combo Kit**	Molecular biology	ThermoFisher/ Invitrogen	K220001
**TRIzol,Reagent**	Molecular biology	ThermoFisher/Invitrogen	15596026
**1.5 ml Eppendorf tubes**	Plasticware	Milian	390903
**2 ml Screwcap Tubes Sarstedt**	Plasticware	SAP	9107130
**50ML BIGPREP LYSING MATRIX B**	Plasticware	MPBio	116951010
**Centrifugation tubes 50ml Polyprpylen ClearLine**	Plasticware	Milian	131032C
**CLEAR-LOCK ClearLine¬Æ - 2 ml**	Plasticware	Milian	390904
**Disposable Reagent reservoirs economy, PS LLG-Labware 25ml**	Plasticware	Huberlab	600.2821.45
**Disposable Reagent reservoirs economy, PS LLG-Labware 50ml**	Plasticware	Huberlab	600.2821.47
**Duran¬Æ baffled flask**	Plasticware	Merck	Z680966-4EA
**Electroporation Cuvettes 2 mm**	Plasticware	FisherScientific	15542423
**Glass test tube 15 ml**	Plasticware	Milian	110034
**Glass test tube lids**	Plasticware	Milian	391597
**Greiner 15 ml tubes blue lid**	Plasticware	Huberlab	7.188 271
**Greiner Microplates 96 Well Polystyrene F-Form**	Plasticware	Huberlab	7.655 161
**Greiner Microplates 96 Well Polystyrene U-Form**	Plasticware	Huberlab	7.650 101
**GREINER-1536-Well Microplates black Cycloolefin SCREENSTAR**	Plasticware	Huberlab	7.789 866
**GREINER-1536-Well SCREENSTAR Polystyrene Lids**	Plasticware	Huberlab	7.691 161
**Lid for 96-well Microplate - sterile**	Plasticware	Milian	390970
**Parafilm 10 cm x 38 m**	Plasticware	FisherScientific	11772644
**Sartorius Minisart NML Syringe filter, sterile, 1.2 um**	Plasticware	FisherScientific	10189220
**Sartorius Minisart NML Syringe filter, sterile, 5.0 um**	Plasticware	FisherScientific	10230891
**Syringe 10ml Sterile**	Plasticware	SAP	1134990
**Syringe 20ml Sterile**	Plasticware	SAP	1135000
**Syringe Sterile Filter 0.22 um Clearline**	Plasticware	Milian	51732
**TREFF - Deep Well Plate 2.2 ml unsteril, PP**	Plasticware	Huberlab	2.9799.01

Supplementary Table 4. Consumables and materials used.
