# Supplementary material for: Large-scale testing of antimicrobial lethality at single-cell resolution predicts mycobacterial infection outcomes
Source: Nat Microbiol. 2026 Jan 9;11(2):566–83. doi: 10.1038/s41564-025-02217-y (PMC12872448; doi:10.1038/s41564-025-02217-y)
Supplement: Supplementary file 2 — Reporting Summary [file 41564_2025_2217_MOESM2_ESM.pdf]

nature portfolio

Corresponding author(s): Lucas Boeck

Last updated by author(s): 5/11/2025

Reporting Summary

Nature Portfolio wishes to improve the reproducibility of the work that we publish. This form provides structure for consistency and transparency in reporting. For further information on Nature Portfolio policies, see our Editorial Policies and the Editorial Policy Checklist.

Statistics

For all statistical analyses, confirm that the following items are present in the figure legend, table legend, main text, or Methods section.

n/a	Confirmed	
☐	☒	The exact sample size (*n*) for each experimental group/condition, given as a discrete number and unit of measurement
☐	☒	A statement on whether measurements were taken from distinct samples or whether the same sample was measured repeatedly
☐	☒	The statistical test(s) used AND whether they are one- or two-sided<br>*Only common tests should be described solely by name; describe more complex techniques in the Methods section.*
☒	☐	A description of all covariates tested
☐	☒	A description of any assumptions or corrections, such as tests of normality and adjustment for multiple comparisons
☐	☒	A full description of the statistical parameters including central tendency (e.g. means) or other basic estimates (e.g. regression coefficient) AND variation (e.g. standard deviation) or associated estimates of uncertainty (e.g. confidence intervals)
☐	☒	For null hypothesis testing, the test statistic (e.g. *F*, *t*, *r*) with confidence intervals, effect sizes, degrees of freedom and *P* value noted<br>*Give P values as exact values whenever suitable.*
☒	☐	For Bayesian analysis, information on the choice of priors and Markov chain Monte Carlo settings
☐	☒	For hierarchical and complex designs, identification of the appropriate level for tests and full reporting of outcomes
☐	☒	Estimates of effect sizes (e.g. Cohen's *d*, Pearson's *r*), indicating how they were calculated

Our web collection on statistics for biologists contains articles on many of the points above.

Software and code

Policy information about availability of computer code

Data collection	No software was used for data collection.
Data analysis	BaSiC v1, Bcftools v1.14, FastTree v2.1, Fiji v2.9.9, Genome Analysis Toolkit v4.2, Graph Pad Prism v10.1.1, Ilastik version v1.3.3, Itol v5, Matlab version, Python v3, Pyseer v1.3.11, R v4.1.2, R caret package v6.0.94, Sambamba v1.0, SNPeffects v4.3.1. All original code is deposited at GitHub (https://github.com/BoeckLab/ASCT).

For manuscripts utilizing custom algorithms or software that are central to the research but not yet described in published literature, software must be made available to editors and reviewers. We strongly encourage code deposition in a community repository (e.g. GitHub). See the Nature Portfolio guidelines for submitting code & software for further information.

Data

Policy information about availability of data

All manuscripts must include a data availability statement. This statement should provide the following information, where applicable:

- Accession codes, unique identifiers, or web links for publicly available datasets
- A description of any restrictions on data availability
- For clinical datasets or third party data, please ensure that the statement adheres to our policy

All sequencing data of this study is deposited at the European Nucleotide Archive (ENA) with respective accession codes provided in source data. Raw imaging data

reported in this study cannot be deposited in a public repository due to size constraints, but is available from the lead contact upon request. All downstream imaging data and all other data are provided with this paper.

Research involving human participants, their data, or biological material

Policy information about studies with human participants or human data. See also policy information about sex, gender (identity/presentation), and sexual orientation and race, ethnicity and racism.

Reporting on sex and gender	N/A
Reporting on race, ethnicity, or other socially relevant groupings	N/A
Population characteristics	405 patients with Mycobacterium abscessus pulmonary infection.
Recruitment	Retrospective clinical metatdata of patients assessed during routine clinical assessments was used. No patient was recruited for this study.
Ethics oversight	Ethical approval to use clinical metadata was obtained from the National Research Ethics Service (NRES; REC reference: 12/EE/0158) and the National Information Governance Board (NIGB; ECC 3-03 (f)/2012) for centres in England and Wales; from NHS Scotland Multiple Board Caldicott Guardian Approval (NHS Tayside AR/SW) for Scottish centres; and respective review boards from Queensland (Australia) and the University of North Carolina (USA).

Note that full information on the approval of the study protocol must also be provided in the manuscript.

Field-specific reporting

Please select the one below that is the best fit for your research. If you are not sure, read the appropriate sections before making your selection.

☒ Life sciences ☐ Behavioural & social sciences ☐ Ecological, evolutionary & environmental sciences

For a reference copy of the document with all sections, see nature.com/documents/nr-reporting-summary-flat.pdf

Life sciences study design

All studies must disclose on these points even when the disclosure is negative.

Sample size	This was a retrospective study. The sample size was based on available clinical samples.
Data exclusions	Replicates of M. abscessus time-kill kinetics not fulfilling the quality criteria were excluded (as outlined in the methods).
Replication	All experiments were assessed in replicates and all replicates analysed, except M. abscessus time-kill kinetics not fulfilling the quality criteria were excluded (as outlined in the methods).
Randomization	N/A. Samples were not allocated to experimental groups.
Blinding	N/A. Samples were not allocated to experimental groups.

Reporting for specific materials, systems and methods

We require information from authors about some types of materials, experimental systems and methods used in many studies. Here, indicate whether each material, system or method listed is relevant to your study. If you are not sure if a list item applies to your research, read the appropriate section before selecting a response.

Materials & experimental systems

n/a	Involved in the study	
☒	☐	Antibodies
☒	☐	Eukaryotic cell lines
☒	☐	Palaeontology and archaeology
☒	☐	Animals and other organisms
☐	☒	Clinical data
☒	☐	Dual use research of concern
☒	☐	Plants

Methods

n/a	Involved in the study	
☒	☐	ChIP-seq
☒	☐	Flow cytometry
☒	☐	MRI-based neuroimaging

Clinical data

Policy information about clinical studies

All manuscripts should comply with the ICMJE guidelines for publication of clinical research and a completed CONSORT checklist must be included with all submissions.

Clinical trial registration	Retrospective observational study.
Study protocol	This was a retrospective study done with previously obtained clinical samples and metadata.
Data collection	Retrospective clinical metatdata of patients assessed during routine clinical assessments was used.
Outcomes	Pulmonary M. abscessus infections were considered cleared, in case of sustained M. abscessus culture conversion after six months of treatment. Otherwise, the M. abscessus infection was considered persistent.

Plants

Seed stocks	N/A
Novel plant genotypes	N/A
Authentication	N/A
